# Integrons and Efflux Pump‐Mediated Antibiotic Resistance in Multidrug‐Resistant (MDR) Uropathogenic *Escherichia coli*

**DOI:** 10.1155/bmri/2158306

**Published:** 2026-04-02

**Authors:** Mohammad Amin Gonbadi, Yasaman Dini, Mohsen Mohammadi, Abazar Pournajaf, Mehrdad Halaji

**Affiliations:** ^1^ Student Research Committee, Babol University of Medical Sciences, Babol, Iran, mubabol.ac.ir; ^2^ Non-Communicable Pediatric Diseases Research Center, Health Research Institute, Babol University of Medical Sciences, Babol, I.R., Iran, mubabol.ac.ir; ^3^ Infectious Diseases and Tropical Medicine Research Center, Health Research Institute, Babol University of Medical Sciences, Babol, I.R., Iran, mubabol.ac.ir

**Keywords:** antibiotic resistance, efflux pumps, integrons, multidrug resistance (MDR), uropathogenic *Escherichia coli* (UPEC)

## Abstract

**Objective:**

Integrons facilitate the acquisition and expression of antibiotic resistance genes, with Classes 1 and 2 commonly found in clinical *E. coli*. The AcrAB–TolC efflux pump is a major RND system that expels diverse antimicrobial agents and contributes to resistance. This study is aimed at determining the prevalence of Class 1/2 integrons and the AcrAB–TolC efflux pump in UPEC isolates from UTI patients in Babol, Iran.

**Methods:**

This cross‐sectional study analyzed 155 UPEC isolates collected from UTI patients in Babol, northern Iran (2021–2023). Isolates were identified using standard biochemical and genotypic methods. Antimicrobial susceptibility was assessed by disk diffusion. Polymerase chain reaction (PCR) was used to detect the presence of integron and efflux pump genes. Statistical analysis was performed using SPSS (Version 16), with significance defined at *p* value < 0.05.

**Results:**

Findings revealed that 85.2% of the isolates carried Class 1 integrons, whereas 23.2% contained Class 2 integrons. Integron‐positive isolates presented an increased level of antibiotic resistance, particularly to trimethoprim–sulfamethoxazole (75% and 88.9%) and nalidixic acid (90.9% and 91.7%). Additionally, over 88% of the isolates had AcrAB–TolC efflux pump genes. MDR rates were 75% among *intI1*‐positive isolates and 83.3% among *intI2*‐positive isolates.

**Conclusion:**

In conclusion, integrons contribute to the spread of antibiotic resistance among UPEC isolates, whereas the AcrAB–TolC efflux pump contributes to the ability of bacteria to expel toxic substances. There is a need for effective infection management policies and proper application of antibiotics to limit the dissemination of resistance genes.

## 1. Introduction

Urinary tract infections (UTIs) can be defined as an inflammation of the urethra (urethritis), bladder (cystitis), or kidney (pyelonephritis) [1, 2]. UTIs represent an important public health concern, influencing more than 150 million people globally and leading to substantial economic consequences, morbidity, and mortality. These infections can relapse and recur in individuals with no apparent structural or functional dysfunctions of the urinary tract or in those who are not undergoing urinary catheterization [1, 3].

Although several bacteria can cause UTIs, the predominant etiological agent is UPEC, a strain from the *Enterobacteriaceae* family. UPEC accounts for approximately 80% of uncomplicated UTIs and 95% of both hospital‐ and community‐acquired infections [4, 5].

The rate of multidrug‐resistant (MDR) UPEC strains varies between developed and developing countries [6, 7]. In Iran, a developing country, the percentage of MDR UPEC strains is estimated to be 49.4% [8]. Antibiotic overuse for UTI treatment has increased antimicrobial resistance in UPEC. The increasing prevalence of MDR UPEC has emerged as a significant clinical challenge in recent decades [9–12]. Resistance genes can be found on plasmids or transposons and transferred between cells through conjugation, transformation, or transduction. This gene transfer allows resistance to spread rapidly within bacterial populations and across different bacterial species [13, 14].

Integrons, which are DNA elements that can capture and express resistance genes via site‐specific recombination, contribute to the dissemination, acquisition, and expression of antibiotic resistance genes among bacteria [15]. Integrons can be categorized into four classes considering the integrase gene, with Classes I (*intI1*) and II (*intI2*) integrons being more prevalent in clinical strains of *Escherichia coli* [16].

Another key mechanism of bacterial antibiotic resistance is the action of efflux pumps, which prevent the assembly of antimicrobial agents within bacterial cells. In *E. coli*, various efflux systems transport drugs and other substrates out of the cell. These systems are categorized into seven major superfamilies: (i) multidrug and toxic compound extrusion (MATE); (ii) resistance‐nodulation‐cell division (RND); (iii) major facilitator (MFS); (iv) proteobacterial antimicrobial compound efflux (PACE); (v) ATP‐binding cassette (ABC); (vi) p‐aminobenzoyl‐glutamate transporter (ABGT); and (vii) small multidrug resistance (SMR) [17, 18]. The AcrAB–TolC system, which belongs to the RND family, serves as the primary efflux pump in *E. coli* isolates and has three components: an outer membrane channel (TolC), a periplasmic membrane fusion protein (AcrA), and an inner membrane transporter (AcrB). These components work to expel several antimicrobial agents, such as organic solvents, antibiotics, detergents, and dyes, from within the bacterial cell [19, 20].

Given the significant effects of efflux pumps and integrons on bacterial antibiotic resistance, as well as the limited research on this topic in our area, we investigated the rates of Classes 1 and 2 integrons and the AcrAB–TolC efflux pump in UPEC isolates from patients with UTIs in considered teaching hospitals in Babol, Iran.

## 2. Materials and Methods

### 2.1. Bacterial Isolates, Study Population, and Identification

This cross‐sectional study was conducted on 155 nonduplicate UPEC isolates from patients with UTIs from 2021 to 2023. The research population consisted of patients hospitalized in Babol, which is located in northern Iran. The isolates were identified via standard biochemical tests, and their characteristics were previously documented in a related study by our team [6]. The isolates were initially cultured on eosin methylene blue (EMB) agar, and colonies with the characteristic metallic green sheen were selected. Confirmation of *E. coli* was performed using standard microbiological and biochemical assays, including indole production, motility at 37°C, nitrate reduction, citrate utilization, urease activity, and MR‐VP tests [21]. In this study, we did not involve sample collection, and instead utilized *E. coli* isolates obtained from urine culture samples that were routinely sent to the laboratory for diagnostic purposes according to standard clinical protocols.

### 2.2. Antimicrobial Susceptibility Testing

The antibiotic resistance profiles of UPEC isolates were assessed via the disk diffusion method on Mueller–Hinton agar plates (Merck, Germany) in accordance with the Clinical and Laboratory Standards Institute (CLSI) instructions. The tested antimicrobial agents included amikacin (AN), ciprofloxacin (CP), nalidixic acid (NA), trimethoprim–sulfamethoxazole (SXT), cefotaxime (CTX), gentamicin (GM), ceftriaxone (CRO), nitrofurantoin (FM), and imipenem (IPM). *E coli* ATCC 25922 was considered the quality control strain for these susceptibility tests [22]. MDR was defined as resistance to at least one antimicrobial agent in at least three different antimicrobial categories [23].

### 2.3. Screening of the ESBL Phenotype

The double‐disk synergy test detected ESBL‐producing *E. coli* isolates. This test employed ceftazidime–clavulanic and CTX–clavulanic acids in combination and compared their effects to those of CTX and ceftazidime individually. A bacterium was classified as an ESBL producer if the inhibition zone surrounding the clavulanic acid discs was at least 5 mm greater than that without it.

### 2.4. Molecular Analysis

Bacterial DNA was isolated from fresh colonies following established methods. The concentration and purity of the extracted DNA were assessed using a NanoDrop spectrophotometer (Thermo Scientific, Wilmington, United States). Polymerase chain reaction (PCR) employs certain primers to identify the existence of the *intI2*, *intI1*, *acrB*, *acrA*, and *tolC* genes.

PCR amplification was performed in a total reaction volume of 12 *μ*L, consisting of 5 *μ*L of ready‐to‐use MasterMix (AMPLIQON, Denmark), 5.2 *μ*L of DNase‐free distilled water, 0.3 *μ*L of each primer (10 pmol/*μ*L), and 1.2 *μ*L of template DNA. PCR was performed using a BOECO Thermal Cycler TC‐TE under the following conditions: an initial denaturation at 94°C for 5 min, followed by 35 cycles of denaturation at 94°C for 45 s, primer annealing at 55°C for *intI1* and *intI2* or 50°C for *tolC*, *acrA*, and *acrB* for (insert time if available, e.g., 45 s), and extension at 72°C for 45 s. A final extension was performed at 72°C for 5 min. The amplified products were analyzed via electrophoresis on 1% agarose gels prepared with 1× EDTA/acetate/Tris buffer, stained with a SafeStain loading dye (CinnaGen Co., Iran), and visualized via UV light.

### 2.5. Statistical Analysis

Continuous and categorical data are presented as the means ± standard deviations (SDs) and frequencies and percentages, respectively. The groups were compared via the chi‐square test. SPSS 16 software, Version 16 (IBM Corp., United States) was used for statistical analysis. *p* value < 0.05 was used to declare statistical significance.

## 3. Results

A total of 155 UPEC isolates were collected from the urine samples of patients. Among the isolated samples, with an age range of less than 1 year to 80 years, the percentages of males and females were 25.8% (40/155) and 74.2% (115/155), respectively. PCR amplification of two classes of integron genes revealed that 85.2% of the isolates (132/155) had *intI1*, 23.2% of the isolates (36/155) had *intI2*, and 23.2% of the isolates (36/155) had both genes.

An increased resistance level in Classes 1 and 2 integron‐positive isolates was observed for SXT (75% and 88.9%, respectively) and NA (90.9% and 91.7%, respectively). In contrast, FM and meropenem were the most effective for integron‐positive isolates. MDR rates were 75% among *intI1*‐positive isolates and 83.3% among *intI2*‐positive isolates.

The complete antibiotic resistance profiles of the integron‐negative and integron‐positive isolates are shown in Tables 1 and 2. The results revealed a significant relationship between the existence of Class I integrons and increased resistance to meropenem and ceftazidime. Similarly, Class II integrons were significantly associated with higher resistance rates against SXT and AN (*p* value ≤ 0.05). PCR analysis revealed that the genes *tolC*, *acrB*, and *acrA* were detected in 96.1%, 92.3%, and 88.4% of the isolates, respectively. Furthermore, *acrB*, *acrA*, and *tolC* were concurrently identified in 88.4% of the isolates.

**Table 1:** Antibiotic susceptibility pattern of Class 1 integron‐positive and integron‐negative of uropathogenic *Escherichia coli* strains.

Antibiotics		Integron‐1 positive *n* = 132 no. (%)	Integron‐1 negative *n* = 23 no. (%)	*p*
Meropenem	R	22 (16.7)	0	0.01
	I	11 (8.3)	1 (4.3)	
	S	99 (75)	22 (95.7)	
Nitrofurantoin	R	16 (12.1)	1(4.3)	0.4
	I	3 (2.3)	0	
	S	113 (85.6)	22 (95.7)	
Cefotaxime	R	91 (68.9)	20 (87)	0.1
	I	3 (2.3)	0	
	S	38 (28.8)	3 (13)	
Ceftriaxone	R	64 (48.5)	15 (65.2)	0.1
	I	0	0	
	S	68 (51.5)	8 (34.8)	
Ceftazidime	R	91 (68.9)	20 (87)	0.05
	I	0	0	
	S	41 (31.1)	3 (13)	
Cefixime	R	57 (43.2)	12 (52.2)	0.5
	I	4 (3)	0	
	S	71 (53.8)	11 (47.8)	
Gentamicin	R	27 (20.5)	4 (17.4)	0.8
	I	1 (0.8)	0	
	S	104 (78.8)	19 (82.6)	
Amikacin	R	28 (21.2)	5 (21.7)	0.3
	I	12 (9.1)	0	
	S	92 (69.7)	18 (78.3)	
Trimethoprim–sulfamethoxazole	R	99 (75)	16 (69.6)	0.6
	I	0	0	
	S	33 (25)	7 (30.4)	
Nalidixic acid	R	120 (90.9)	22 (90.7)	0.6
	I	0	0	
	S	12 (9.1)	1 (4.3)	
Norfloxacin	R	82 (62.1)	10 (43.5)	0.1
	I	0	0	
	S	50 (37.9)	13 (56.5)	
Ciprofloxacin	R	90 (68.2)	13 (56.5)	0.3
	I	0	0	
	S	42 (31.8)	10 (43.5)	
Ofloxacin	R	64 (48.5)	11 (47.8)	1
	I	0	0	
	S	68 (51.5)	12 (52.2)	
Amoxicillin/clavulanic acid	R	59 (44.7)	8 (34.8)	0.4
	I	22 (16.7)	3 (13)	
	S	51 (38.6)	12 (52.2)	
Ampicillin–sulbactam	R	83 (62.9)	17 (73.9)	0.4
	I	5 (3.8)	0	
	S	44 (33.3)	6 (26.1)	
MDR	P	99 (75)	16 (69.6)	0.6
	N	33 (25)	7 (30.4)	
ESBL	P	86 (65.2)	19 (82.6)	0.1
	N	46 (34.8)	4 (17.4)	

**Table 2:** Antibiotic susceptibility pattern of Class 2 integron‐positive and integron‐negative of uropathogenic *Escherichia coli* strains.

Antibiotics		Integron‐2 positive *n* = 36 no. (%)	Integron‐2 negative *n* = 119 no. (%)	*p*
Meropenem	R	1 (2.8)	21 (17.6)	0.05
	I	2 (5.6)	10 (8.4)	
	S	33 (91.7)	88 (73.9)	
Nitrofurantoin	R	1 (2.8)	16 (13.4)	0.11
	I	0	3 (2.5)	
	S	35 (97.2)	100 (84)	
Cefotaxime	R	28 (77.8)	83 (69.7)	0.52
	I	1 (2.8)	2 (1.7)	
	S	7 (19.4)	34 (28.6)	
Ceftriaxone	R	14 (38.9)	65 (54.6)	0.12
	I	0	0	
	S	22 (61.1)	54 (45.4)	
Ceftazidime	R	28 (77.8)	83 (69.7)	0.4
	I	0	0	
	S	8 (22.2)	36 (30.3)	
Cefixime	R	16 (44.4)	53 (44.5)	0.99
	I	1 (2.8)	3 (2.5)	
	S	19 (52.8)	63 (52.9)	
Gentamicin	R	4 (11.1)	27 (22.7)	0.26
	I	0	1 (0.8)	
	S	32 (88.9)	91 (76.5)	
Amikacin	R	11 (30.6)	22 (18.5)	0.05
	I	5 (13.9)	7 (5.9)	
	S	20 (55.6)	90 (75.6)	
Trimethoprim–sulfamethoxazole	R	32 (88.9)	83 (69.7)	0.02
	I	0	0	
	S	4 (11.1)	36 (30.3)	
Nalidixic acid	R	33 (91.7)	109 (91.6)	1
	I	0	0	
	S	3 (8.3)	10 (8.4)	
Norfloxacin	R	20 (20.5)	72 (60.5)	0.69
	I	0	0	
	S	47 (36.5)	47 (39.5)	
Ciprofloxacin	R	20 (55.6)	83 (69.7)	0.15
	I	0	0	
	S	16 (44.4)	36 (30.3)	
Ofloxacin	R	17 (47.2)	58 (48.7)	1
	I	0	0	
	S	19 (52.8)	61 (51.3)	
Amoxicillin/clavulanic acid	R	14 (38.9)	50 (44.5)	0.2
	I	11 (30.6)	14 (11.8)	
	S	11 (30.6)	52 (43.7)	
Ampicillin–sulbactam	R	22 (61.1)	78 (65.5)	0.67
	I	2 (5.6)	3 (2.5)	
	S	12 (33.3)	38 (31.9)	
MDR	P	30 (83.3)	85 (71.4)	0.19
	N	6 (16.7)	34 (28.6)	
ESBL	P	28 (77.8)	77 (64.7)	0.15
	N	8 (22.2)	42 (53.3)	

Abbreviations: I, intermediate; R, resistance; S, susceptible.

## 4. Discussion

This study demonstrates the overall prevalence and distribution of integrons in UPEC isolates from Babol, northern Iran. We revealed a significant genetic mechanism underlying antimicrobial resistance, whereby integrons harbor and disseminate resistance genes in the most common human pathogen, *E. coli*. In this study, 85.2% of the isolates harbored Class 1 integrons, 23.2% harbored Class 2 integrons, and 23.2% contained both classes. The increased prevalence of *intI1* and *intI2* in our region raises significant concerns for the future, as it may cause the spread and growth of antibiotic resistance associated with Classes 1 and 2 integrons.

The occurrence and features of integrons in *E. coli* isolates can differ on the basis of factors such as geographical region, the year of isolation, and the infection site. A meta‐analysis covering studies conducted up to July 2019 from multiple countries revealed that the worldwide incidence of Class 1 integrons in *E. coli* isolates was 47%, with reported rates ranging from 6% to 90% [24]. In a 2024 study from Denizli, Turkey, which investigated the distribution and prevalence of integrons in blood culture isolates, 41% and 2% of the isolates carried Classes 1 and 2 integrons, respectively, whereas 2% tested positive for both classes [16]. Similarly, a 2023 study by Mohebi et al. in Iran examined the prevalence of integrons among UPEC isolates. Integrons were identified in 69 (67.6%) strains. Among these, 59 isolates had only Class 1 integrons, four isolates harbored only Class 2 integrons, and six isolates had only Classes 1 and 2 integrons [25]. In 2022, Gumus et al. reported that, among 50 UPEC strains of patients with uncomplicated cystitis and uncomplicated pyelonephritis in Istanbul, 74% of those in Turkey carried Class 1 integrons, 44% carried Class 2 integrons, and 42% possessed both classes [26]. A study conducted in China revealed that integrons are more commonly detected in UPEC isolates than in commensal *E. coli* isolates. Findings from this study, together with previous research, suggest that Class 1 integrons are relatively prevalent in clinical isolates and appear to occur more frequently than in fecal or commensal *E. coli* isolates. The low incidence of integrons reported in certain studies might be attributed to resistance genes on alternative genetic elements, such as transposons and bacteriophages [27].

Alzaidi et al. (2025) found that 22.2% of *E. coli* isolates carried Class 1 integrons, whereas Class 2 and dual Class 1/Class 2 integrons were detected in 6.7% and 3.3% of isolates, respectively. No Class 3 integrons were identified. Their study also showed a significant association between Class 1 integrons and MDR (*p* value < 0.05), whereas integron carriage was not linked to biofilm formation.

MDR and various classes of integrons, particularly Class 1 integrons, are correlated (30). Consistent with our findings, the prevalence of MDR isolates was markedly greater among integron‐positive isolates than among integron‐negative isolates, underscoring the vital role of integrons in the spread of resistance genes among pathogens [25]. Our antibiotic susceptibility analysis revealed a significant link between the existence of Classes 1 and 2 integrons and increased resistance to meropenem, ceftazidime, SXT, and AN, as opposed to integron‐negative isolates. In support of our results, Mohebi et al. [25] reported an increased rate of MDR isolates among integron‐positive strains. Similarly, Oner et al. [28] reported that integron‐positive strains presented elevated MDR rates, with notable resistance to ampicillin and SXT. Additionally, Shariat et al. [13] reported a significant correlation between the existence of Classes 1, 2, and 3 integrons and resistance to AN, SXT, CP, GM, and NA.

In this study, 88.4%, 92.3%, and 96.1% of the isolates contained the *acrB*, *acrA*, and *tolC* genes, respectively. Furthermore, *acrB*, *acrA*, and *tolC* were found together in 88.4% of the isolates. Fayyazi et al. [24] reported that the *acrA*, *acrB*, and *tolC* genes were present in 78.4% (87/111), 89.2% (99/111), and 82.9% (92/111) of the isolates, respectively. Additionally, 68.5% of UPEC isolates in their study possessed all three genes simultaneously. In another study, Gawad et al. [20] reported that 74.3% of UPEC isolates harbored the *tolC, acrA*, and *acrB* genes concurrently. Similarly, Ganjo et al. reported that the *acrA* and *acrB* genes represented the most frequent gene combination among the isolates (20%). They also observed a significant association between the presence of the *acrA* gene and increased resistance to levofloxacin (*p* value = 0.022). Furthermore, the *acrB* gene was significantly correlated with decreased resistance to meropenem (*p* value = 0.006) and increased resistance to levofloxacin (*p* value = 0.036). These findings underscore the role of efflux pump components in modulating antimicrobial susceptibility patterns in UPEC isolates [29].

We did not find a significant association between the existence of the *acrB*, *acrA*, and *tolC* genes and resistance to antibiotics. A limitation of the present study is that the detection of efflux‐related genes does not necessarily reflect their functional activity. Additionally, we did not assess the clinical implications of the observed resistance patterns or gene profiles, which represent another limitation of our work.

## 5. Conclusion

In conclusion, our findings underscore the pivotal role of Classes 1 and 2 integrons in the emergence and dissemination of MDR, particularly among UPEC isolates. These integrons are significantly associated with resistance against meropenem, cotrimoxazole, ceftazidime, and AN, facilitating the acquisition and horizontal transfer of antibiotic resistance genes. Additionally, RND efflux pumps, notably AcrAB–TolC, contribute to antibiotic resistance in gram‐negative bacteria by expelling toxic materials, such as antibiotics.

## Author Contributions

**Methodology:** Yasaman Dini, Mohammad Amin Gonbadi, and Mehrdad Halaji; **supervision**: Mohsen Mohammadi, Abazar Pournajaf, and Mehrdad Halaji; **writing—original draft:** Yasaman Dini, Mohammad Amin Gonbadi, Mohsen Mohammadi, Abazar Pournajaf, and Mehrdad Halaji; **formal analysis:** Abazar Pournajaf and Mohammad Amin Gonbadi.

## Funding

No funding was received for this manuscript.

## Ethics Statement

The study was approved by the Research Ethics Committee of Babol University of Medical Sciences (No. IR.MUBABOL.REC.1402.072), Babol, Iran. In this study, bacteria isolated from clinical samples in the clinical microbiology laboratory were used.

## Consent

The study utilized microbial plates from routine laboratory diagnostics. These samples were collected as part of standard patient care, and no additional samples were required. All data were anonymized to ensure patient privacy.

## Conflicts of Interest

The authors declare no conflicts of interest.

## Data Availability

The data that support the findings of this study are available from the corresponding author upon reasonable request.
